# Monocyte-Derived Dendritic Cells Can Revert In Vitro Antigen-Specific Cellular Anergy in Active Human Paracoccidioidomycosis

**DOI:** 10.3390/jof7030201

**Published:** 2021-03-10

**Authors:** Paula Keiko Sato, Telma Miyuki Oshiro, Érika Cano Passos, Tatiana Giselle Rodrigues Miranda, Constância Lima Diogo, Claudia de Abreu Fonseca, Aya Sadahiro, Sandro Rogério de Almeida, Maria Aparecida Shikanai-Yasuda

**Affiliations:** 1 Departament of Infectious Diseases, Faculdade de Medicina FMUSP, Universidade de Sao Paulo, Sao Paulo 05403-000, Brazil; tmoshiro@yahoo.com (T.M.O.); tati.biomedica@hotmail.com (T.G.R.M.); ayasadahiro@gmail.com (A.S.); 2 Laboratory of Medical Investigation in Immunology (LIM-48), Hospital das Clinicas HCFMUSP, Faculdade de Medicina, Universidade de Sao Paulo, Sao Paulo 05403-000, Brazil; erikafootstep@yahoo.com.br (É.C.P.); constanciadl@yahoo.com.br (C.L.D.); c.lau.2007@hotmail.com (C.d.A.F.); 3 Institute of Tropical Medicine, Faculdade de Medicina FMUSP, Universidade de Sao Paulo, Sao Paulo 05403-000, Brazil; 4 Laboratory of Medical Investigation in Dermatology and Immunodeficiencies (LIM-56), Hospital das Clinicas HCFMUSP, Faculdade de Medicina, Universidade de Sao Paulo, Sao Paulo 05403-000, Brazil; 5 Departament of Parasitology, Instituto de Ciências Biológicas, Universidade Federal do Amazonas, Manaus 69080-900, Brazil; 6 Department of Clinical and Toxicological Analysis, Faculty of Pharmaceutical Sciences, Universidade de Sao Paulo, Sao Paulo 05508-000, Brazil; sandroal@usp.br

**Keywords:** *Paracoccidioides brasiliensis*, paracoccidioidomycosis, dendritic cells, cytokines, coculture, gp43, cell-free antigen

## Abstract

We investigated the in vitro effects of two *Paracoccidioides brasiliensis* antigens on monocyte-derived dendritic cells (moDCs) from patients with paracoccidioidomycosis (PCM). MoDCs from patients with active or treated PCM and non-PCM subjects were generated, stimulated with TNF-α, and *P. brasiliensis* antigens, 43 kDa glycoprotein (gp43) and cell-free antigen (CFA), and analyzed by flow cytometry and enzyme-linked immunosorbent assays (ELISA). Our data revealed that patients with PCM had a high frequency of HLA-DR^+^ cells, but the treated group had more CD86^+^ cells with increased IL-12p40. Patients with active PCM had more CD80^+^ moDCs, and as a novel finding, large amounts of chemokine (C-C motif) ligand 18 (CCL18) in the supernatants from their in vitro moDC cultures. Both gp43- and CFA-stimulated moDCs from the patients with PCM successfully reverted the in vitro antigen-specific anergy, inducing a proliferative response. However, CFA-stimulated moDCs led to higher lymphoproliferation, with increased IFN-γ and TNF-α in the cells from the patients with active PCM compared with gp43. These original results combined with constant IL-10 and increased IL-12p40 levels suggest that a more complex antigen, such as CFA, may be a better inducer of the protective Th1 immune response than purified gp43 is, and a suitable target for future studies on anti-*P. brasiliensis* dendritic cell (DC)-based vaccines.

## 1. Introduction

The leading cause of nosocomial mortality among systemic mycosis patients in Brazil is paracoccidioidomycosis (PCM) [1]. This chronic systemic mycosis is endemic in Latin America and caused by dimorphic pathogenic fungal agents from the *Paracoccidioides brasiliensis* (*P. brasiliensis*) complex and *Paracoccidioides lutzii* [2,3,4]. In the lungs, conidia may undergo conversion to infective yeast form leading to a broad spectrum of clinical manifestations. More commonly, the fungus remains in a latent stage resulting in asymptomatic infection [5,6]. Other factors such as virulence of the fungal agent and immune responses may determine the severity of the disease and its clinical forms.

The T-helper (Th)1 immune response with effective axis IFN–γ/IL-12 plays an important protective role in the resistance to *P. brasiliensis* [7,8] and in the asymptomatic PCM infection [9,10]. The chronic form presents a Th17/Th22 profile, with high production of IL-17 and IL-22, also secreting Th1 cytokines such as IFN-γ, TNF-α, IL-2, and variable levels of IL-10 and IL-4, and increased levels of specific IgG1 antibodies, contributing to milder clinical manifestations. The acute form of PCM, in turn, shows a mixed Th2/Th9 response associated with susceptibility: increased levels of IL-4, IL-5, IL-9, IL-10, and TGF-β; low production of IFN-γ and TNF-α; and high levels of specific IgG4 and IgE antibodies [9,10]. Moreover, patients with disseminated PCM present an imbalance in cytokine profile and transitory anergy of the in vitro *P. brasiliensis* antigen-specific cellular immune response during active disease, but recover after successful antifungal treatment [11,12].

Dendritic cells (DCs) are key players in the modulation of those adaptive immune responses, described as professional antigen-presenting cells (APCs) found in the majority of tissues capturing and processing infectious agents. After migrating to peripheral lymph nodes, DCs maturate and express high amounts of costimulatory molecules and the major histocompatibility complex (MHC)-peptide complex, establishing an interaction with T-cells. This immune synapse along with the sort of captured antigen may define the resulting effector T cell response [13,14].

In the murine model of PCM, DCs from resistant mice were shown to be more efficient in inducing lymphocyte proliferation and Th1 cytokines than cells from susceptible animals, which had a preferential response of B cells and macrophages [15,16]. Regardless, DCs from both susceptible and resistant mice showed decreased expression of MHC-II and adhesion molecules, and lower levels of IL-12 when primed with *P. brasiliensis* or gp43 in vitro [17,18].

On cutaneous lesions from patients with PCM, Langerhans cells were shown to be in reduced numbers, and the presence of factor XIIIa+ dermal dendrocytes, dermal DCs and plasmacytoid DCs was also reported [19,20,21,22]. Some reports demonstrated relevant effects of *P. brasiliensis* on monocyte-derived dendritic cells (moDCs) from healthy donors, especially on innate immunity mechanisms [23,24,25,26], but few investigated these cells from patients with PCM [27,28].

Previously, we showed higher expression of Human Leukocyte Antigen—DR isotype (HLA-DR), CD86, and Dendritic Cell-Specific Intercellular adhesion molecule-3-Grabbing Non-integrin (DC-SIGN), and upregulation of IL-12p40 in moDCs from patients with treated PCM compared with in control subjects and patients with the active disease [28]. In the present study, we investigated the effects of two different *P. brasiliensis* antigens on moDCs from patients with active and treated PCM, and we show new perspectives on the modulation of immune responses towards protection and better outcomes.

## 2. Materials and Methods

### 2.1. Paracoccidioidomycosis (PCM) Patients and Control Subjects

In total, 53 patients with PCM diagnosis were included in this study: 24 with active PCM and 29 with treated PCM. All patients were selected at the Systemic Mycosis Outpatient Clinic of the Infectious Diseases Division of Hospital das Clinicas HCFMUSP, Faculdade de Medicina, University of Sao Paulo. Identification of *Paracoccidioides* spp. by mycological, and/or histopathological examination, and/or the presence of anti-*P. brasiliensis* serum antibodies (titers of ≥32 in counterimmunoelectrophoresis test) at the moment of enrollment confirmed the diagnosis of active PCM. Confirmed diagnosis of PCM in the past, the absence of clinical symptoms, and specific antibody titers of ≤4 for a period of at least 6 months determined treated PCM. The control group consisted of 30 non-PCM subjects: individuals considered healthy without a previous history of the disease or detectable anti-*P. brasiliensis* serum antibodies (by immunodiffusion test), and not sensitized in lymphoproliferation assays against the 43 kDa glycoprotein of *P. brasiliensis*. Subjects with comorbidities such as neoplasia and other acute or chronic systemic infectious diseases were excluded. More data about the PCM patients and control subjects can be found in Table S1, Supplementary Material.

### 2.2. P. brasiliensis Antigens

Antigens gp43 [29] and cell-free antigen (CFA) [30] were obtained as previously described. Briefly, gp43 was purified at the Laboratory of Mycology from the Institute of Biomedical Sciences (University of Sao Paulo, Sao Paulo, SP, BR) and at the Laboratory of Medical Investigation in Immunology (HCFMUSP) from the exoantigen of *P. brasiliensis* yeast B-339 strain through an adsorbent column with murine monoclonal antibody anti-gp43 coupled to an Affi-Gel 10 column (Bio-Rad, Hercules, CA, USA). Gp43 was eluted with acid buffer (pH 2.8) and neutralized with 1M Tris (pH 9.0). The material was concentrated in a 10K Amicon apparatus (Merck Millipore, Darmstadt, Germany). CFA was obtained by mixing a suspension of *P. brasiliensis* yeast B-339 strain in RPMI 1640 medium (Gibco, Grand Island, NY, USA) in a vortex mixer and immediately centrifuging at 10,000× *g*. The resulting supernatant fluid contained the antigen. Both gp43 and CFA had their protein contents determined by the Bradford method [31], confirmed by sodium dodecyl sulfate-polyacrylamide gel electrophoresis (SDS-PAGE) [32], and stored at −80 °C until use.

### 2.3. Monocyte-Derived Dendritic Cells (MoDCs) Generation and Stimulation

Monocyte–derived DCs were generated from peripheral blood mononuclear cells (PBMCs) by a modification of previously described methods [33,34]. PBMCs from all subjects were isolated by Ficoll-Hypaque density gradient (GE Healthcare Life Sciences, Uppsala, Sweden) and washed in RPMI 1640 medium (Gibco, Grand Island, NY, USA). Cells (1 × 10^7^/mL) were added to 6-well flat bottom plates (Corning, Steuben, NY, USA) and allowed to adhere for 2 h in a 37 °C incubator with 5% CO_2_. Non-adherent cells were removed by several washes with RPMI 1640 medium (Gibco, Grand Island, NY, USA) and frozen at −80 °C for future use. Adherent cells were cultured for 6 days in RPMI 1640 medium (Gibco, Grand Island, NY, USA) supplemented with gentamicin (40 mg/L), fetal calf serum (10%; Gibco, Grand Island, NY, USA), L-glutamine (2 mol/L; Gibco, Grand Island, NY, USA), recombinant IL-4 (50 ng/mL; PeProtech, Rocky Hill, NJ, USA), and granulocyte–macrophage colony-stimulating factor (GM-CSF, 50 ng/mL; PeProtech, Rocky Hill, NJ, USA). On days 3 and 6, fresh RPMI 1640 medium containing IL-4 and GM-CSF was added to the cultures. On Day 6, cells were harvested and split into 24-well flat bottom plates (1 × 10^6^ cells/mL; Corning, Steuben, NY, USA). MoDCs were then stimulated with gp43 (5 μg/mL) or CFA (15 μg/mL), with or without recombinant TNF-α (50 ng/mL; PeProtech, Rocky Hill, NJ, USA), or left untreated (Medium) for 48 h in a 37 °C incubator with 5% CO_2_.

### 2.4. Flow Cytometric Analyses

The effects of gp43 and CFA on moDC surface molecules expression were investigated by flow cytometry after 48 h of incubation. Specific PE- or FITC-conjugated monoclonal antibodies anti-CD11c, -CD14, -CD1a, -HLA-DR, -CD80, -CD86 (Caltag Medsystems, Buckingham, UK), DC-SIGN (R&D Systems, Minneapolis, MN, USA) and respective mouse isotype controls were used. Cells (1 × 10^6^/mL) were stained for 30 min with manufacturer’s recommended concentrations of mAbs on PBS with fetal calf serum (10%; Gibco, Grand Island, NY, USA) to block any non-specific binding, on ice and in the dark, and then washed 3 times with PBS before acquisition and analysis on a FACSCalibur flow cytometer and CellQuest software (BD Biosciences, San Jose, CA, USA). Results are expressed as percentages of positively stained cells with specific antibodies. Data regarding the gating strategy can be found in Figure S1, Supplementary Material.

### 2.5. Antigen Presentation Assays

PBMCs from PCM and non-PCM control subjects were isolated by Ficoll-Hypaque (GE Healthcare Life Sciences Uppsala, Sweden) density gradient and washed in RPMI 1640 medium (Gibco, Grand Island, NY, USA). Cells (21 × 10^6^/mL) were added to a 96 well flat bottom microplate in triplicate wells (Corning, Steuben, NY, USA) and stimulated with phytohemagglutinin (PHA; 5 µg/mL; Sigma-Aldrich, St. Louis, MO, USA), gp43 (1 µg/mL; determined in a previous study [35]), CFA (2 and 5 µg/mL) or left unstimulated (Medium) at a final volume of 200 µL/well in a 37 °C incubator with 5% CO_2_. Lymphoproliferation was verified on the microplates after 120 h by measuring [^3^H]-thymidine uptake (9.25 × 10^4^ Bq/mL; GE Healthcare Life Sciences, Uppsala, Sweden) during the last 8 h of the assay on a beta plate scintillation counter (Perkin-Elmer, Waltham, MA, USA).

MoDCs were stimulated with *P. brasiliensis* antigens or left untreated as described in Section 2.3. After 48 h, moDCs were irradiated with 15 Gγ from a ^60^Co source and plated at 1 × 10^5^ cells/mL in triplicate wells on 96-well flat-bottom microplates (Corning, Steuben, NY, USA). Autologous non-adherent cells were added at 5 × 10^5^ cells/mL (final volume of 200 µL/well), and microplates were incubated at 37 °C with 5% CO_2_. Triplicate wells with unstimulated moDCs in medium, non-adherent cells in medium and non-adherent cells with PHA 5 µg/mL (Sigma-Aldrich, St. Louis, MO, USA) were used as controls for the assay. The lymphoproliferation was verified after 120 h by measuring [^3^H]-thymidine uptake (9.25 × 10^4^ Bq/mL; GE Healthcare Life Sciences, Uppsala, Sweden) during the last 8 h of the assay on a beta plate scintillation counter (Perkin-Elmer, Waltham, MA, USA).

Results are expressed as the difference between mean counts per minute of triplicates (δ cpm) of stimulated and unstimulated cells (Medium).

### 2.6. Cytokine Analyses

In primary cultures, moDCs were stimulated with *P. brasiliensis* antigens, with or without TNF-α, or left untreated. Culture supernatants were harvested after 48 h of incubation, and cytokines production were assayed by enzyme-linked immunosorbent assay (ELISA) for IL-12p40, IL-1β, IL-10 (BD Biosciences, San Jose, CA, USA), and chemokine (C-C motif) ligand 18 (CCL18) (R&D Systems, Minneapolis, MN, USA), according to manufacturer’s instructions. MoDCs and autologous non-adherent cells were cocultured and supernatants were harvested after 144 h of incubation for cytokines levels detection by ELISA for IFN-γ, TNF-α, IL-4, and IL-10 (BD Biosciences, San Jose, CA, USA) according to the manufacturer’s instructions. IFN-γ levels were also determined on supernatants from the PBMC cultures after 144 h of incubation by ELISA assay (BD Biosciences, San Jose, CA, USA) according to the manufacturer’s instructions.

Results are expressed as picograms per milliliter (pg/mL), except for CCL18, which is expressed as nanograms per milliliter (ng/mL).

### 2.7. Statistical Analyses

Statistical significance of differences between moDCs from the evaluated groups regarding the expression of surface molecules, the induction of lymphoproliferation, or cytokine release was assessed by one-way ANOVA. When values *p* < 0.05 in ANOVA, Bonferroni’s multiple-comparison test was used (all *p* values from ANOVA are detailed in Table S2a–d, Supplementary Material). Results are represented as means with standard error of mean (SEM), and values of *p* < 0.05 were considered statistically significant. All results were analyzed using Graph-Pad Prism 5.01 software (GraphPad Software, San Diego, CA, USA).

## 3. Results

### 3.1. P. brasiliensis Antigens Modulate Surface Molecule Expression on MoDCs from PCM Patients

MoDCs from patients with active and treated PCM, and from non-PCM control subjects were differentiated from monocytes on in vitro cultures. Cells were stimulated with TNF-α or left untreated, and flow cytometric analysis was conducted to investigate the expression of CD14 (monocyte marker), CD11c and CD1a (dendritic cell markers), HLA-DR (MHC class II human antigenic presentation molecule), CD80 and CD86 (costimulatory molecules), and DC-SIGN (C-type lectin receptor). The resulting cells from all groups had morphological characteristics of DCs on optical microscopy observation, and were >80% CD11c^+^CD1a^+^ and <5% CD14^+^ on flow cytometric analysis, indicating successful differentiation of DCs from monocytes. With the addition of TNF-α, these cells were activated, resulting in the increased expression of HLA-DR and CD86 [36].

**Figure 1:**
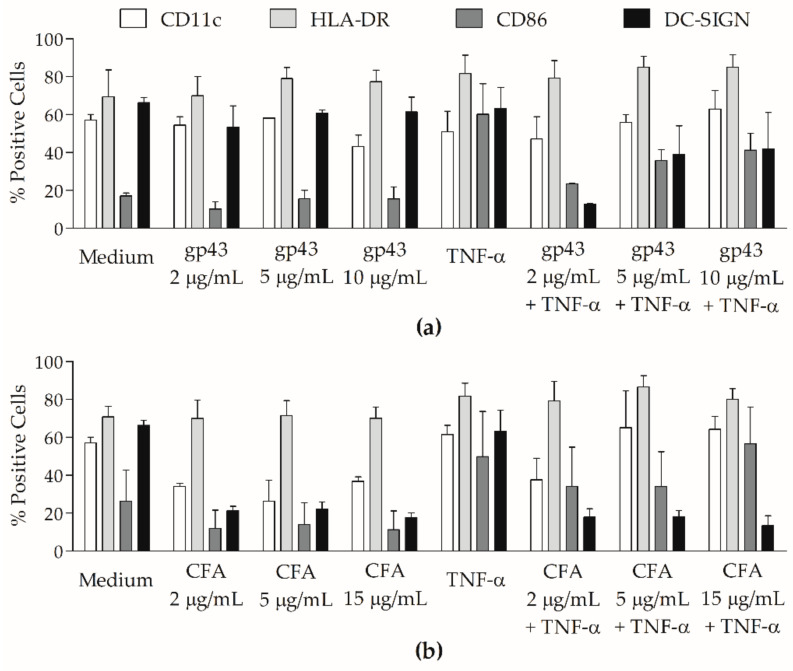
Influence of different concentrations of gp43 and cell-free antigen (CFA) of *P. brasiliensis* on expression of surface molecules by monocyte-derived dendritic cells (moDCs): Percentages of CD11c^+^ (white bars), HLA-DR^+^ (light grey bars), CD86^+^ (dark grey bars) and DC-SIGN^+^ (black bars) cells were analyzed by flow cytometry on moDCs from patients with treated PCM (*n* = 3), after 48 h of stimulation with (**a**) gp43 2, 5 or 10 μg/mL; and (**b**) CFA 2, 5 or 15 μg/mL, with or without TNF-α, or without any treatment (Medium). Results expressed as means with standard errors of the mean (SEM) of percentages of positive cells. There were no statistically significant differences.

We then investigated the effects of gp43 and CFA on the expression of surface molecules on moDCs (Figure 1). We evaluated three different concentrations of each antigen on moDC cultures from patients with treated PCM; gp43 with TNF-α down-regulated CD86 and DC-SIGN when compared with TNF-α alone, and CFA with or without TNF-α decreased DC-SIGN when compared with moDCs and Medium alone or TNF-α alone. Given that gp43 at 5 μg/mL and CFA at 15 μg/mL had more noticeable effects on modifying the percentages of those molecules on gated moDCs (without statistically significant differences), we chose them for further experiments with all groups.

### 3.2. Patients with Active PCM Have More CD80^+^ Cells and CD86^+^ Cells Are Augmented after Antifungal Treatment

**Figure 2:**
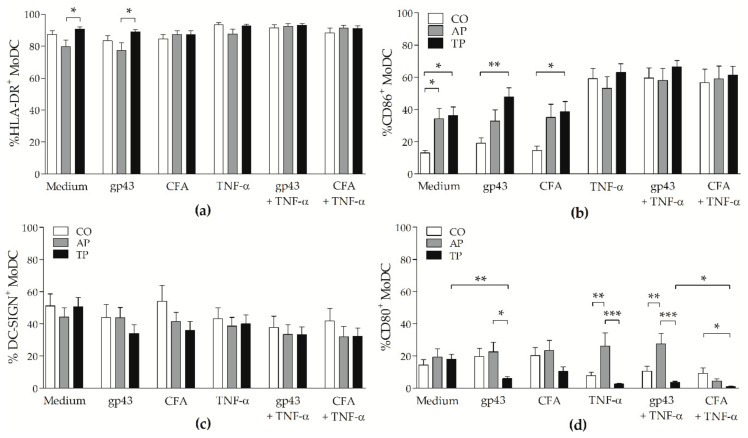
Influence of gp43 and CFA of *P. brasiliensis* on expression of surface molecules by moDCs: Percentages of (**a**) HLA-DR^+^, (**b**) CD86^+^, (**c**) DC-SIGN^+^, and (**d**) CD80^+^ cells analyzed by flow cytometry on gated moDCs from non-PCM control subjects (CO: white bars; *n* = 15), and patients with active PCM (AP: grey bars; *n* = 17) or treated PCM (TP: black bars; *n* = 22), after 48 h of incubation with gp43 or CFA, with or without TNF-α, or left untreated (Medium). Results expressed as means with SEM of percentages; capped lines indicate statistically significant differences with respective *p* values: * *p* < 0.05, ** *p* < 0.01, or *** *p* < 0.001.

After incubation for 48 h, the frequencies of HLA-DR^+^ moDCs from the active PCM group were significantly lower than those of the treated group when cells were stimulated with gp43 or left unstimulated (*p* < 0.05 on both comparisons; Figure 2a). This antigen and TNF-α also increased the mean fluorescence intensity (MFI) of HLA-DR on moDCs from treated patients when compared with cells from the active PCM group (*p* < 0.05; Figure S2a). Interestingly, both groups of PCM patients had higher percentages of CD86^+^ unstimulated cells than non-PCM subjects did (both *p* < 0.05); however, only moDCs from treated patients showed this increase when stimulated with gp43 or CFA (*p* < 0.01 and *p* < 0.05, respectively; Figure 2b). No differences in the number of CD86^+^ cells were observed between the groups in the presence of TNF-α, and frequencies of DC-SIGN^+^ cells were similar in all groups with or without stimuli (Figure 2c). Gp43 significantly decreased the percentages of CD80^+^ cells from the treated PCM group. Conversely, moDCs from the active disease group had higher frequencies of positive cells than those of treated patients in the presence of gp43 with or without TNF-α (Figure 2d).

### 3.3. Patients’ MoDCs Secrete More CCL18 during Active PCM and More IL-12p40 after Antifungal Treatment

**Figure 3:**
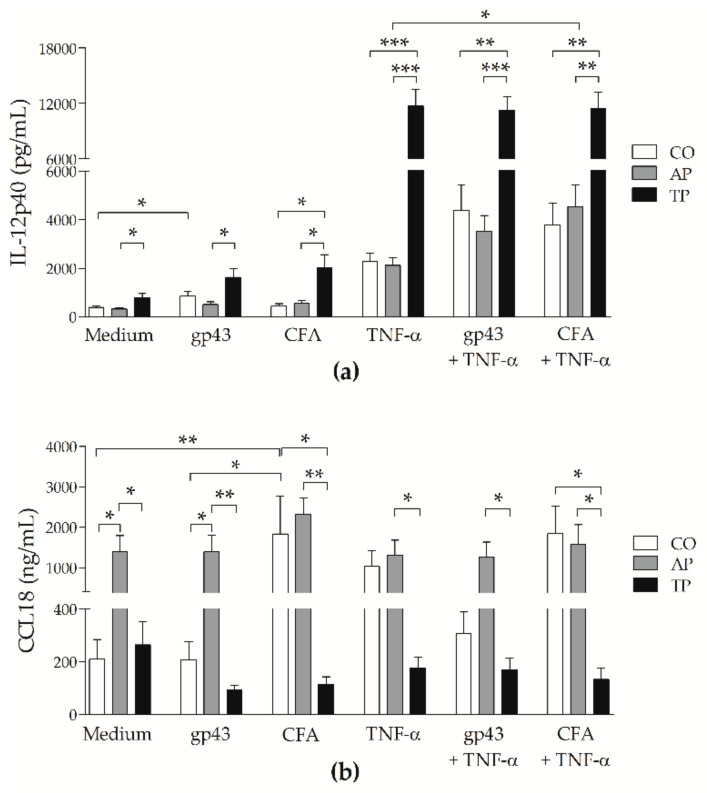
Influence of gp43 and CFA of *P. brasiliensis* on secretion of cytokines by moDCs: Enzyme-linked immunosorbent assayed (ELISA) levels of (**a**) IL-12p40 (pg/mL), and (**b**) CCL18 (ng/mL) measured on moDCs of non-PCM control subjects (CO: white bars; *n* = 15) and patients with active PCM (AP: grey bars; *n* = 17) or treated PCM (TP: black bars; *n* = 22), after 48 h of stimulation with gp43 or CFA, with or without TNF-α, or left untreated (Medium). Results expressed as means with SEM of levels, and capped lines indicate statistically significant differences with respective *p* values: * *p* < 0.05, ** *p* < 0.01 or *** *p* < 0.001.

MoDCs were analyzed by flow cytometry, and the supernatants of those cultures were assayed for IL-12p40, IL-10, IL-1β, and CCL18 by ELISA. Levels of IL-12p40 were significantly higher on moDCs from treated PCM patients when compared with those of the controls or active disease groups, especially with TNF-α (Figure 3a). Supernatants were also assayed for IL-12p70, but there were no detectable levels [36]. MoDCs from both treated and active PCM groups showed levels of IL-10 and IL-1β similar to those from control cells, and there were no statistically significant differences between groups or stimuli [36]. On the other hand, chemokine CCL18 was detected at significantly higher levels in moDC cultures from the active PCM group when compared with the other groups. CFA induced moDCs from the controls to secrete more CCL18 than gp43 or unstimulated cells, and more than CFA-stimulated moDCs from the treated PCM group. In the presence of CFA, with or without TNF-α, moDCs from active PCM patients showed similar levels to those of control subjects, and both groups had higher levels than those of the treated disease group (Figure 3b).

### 3.4. Cell-Free Antigen (CFA) Induces Proliferation on Peripheral Blood Mononuclear Cells (PBMCs) from Patients with Active PCM But Not Stronger than That from Treated Patients

**Figure 4:**
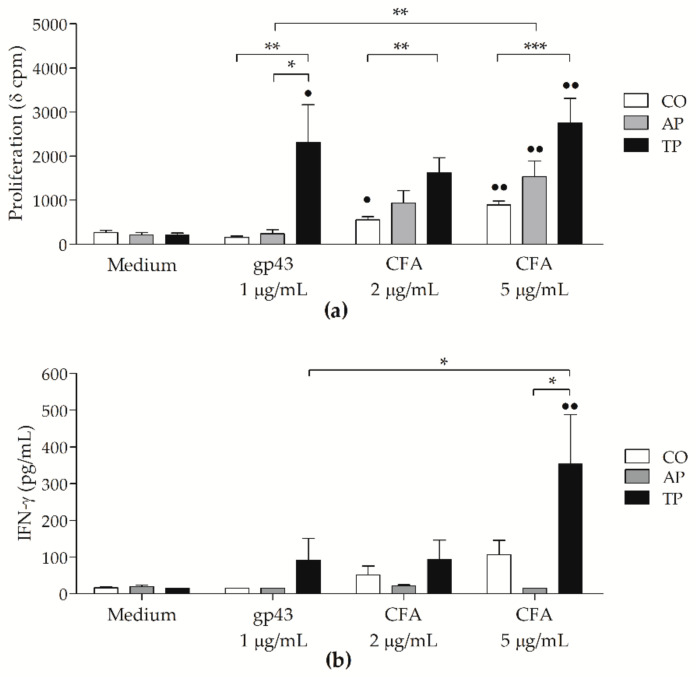
Induction of lymphoproliferation by *P. brasiliensis* antigens on peripheral blood mononuclear cells (PBMCs) culture: (**a**) proliferation of PBMCs was measured by [^3^H]-thymidine uptake on cells; (**b**) levels of IFN-γ (pg/mL) determined on culture supernatants from non-PCM control subjects (CO: white bars; *n* = 17), and patients with active PCM (AP: grey bars; *n* = 08) or treated PCM (TP: black bars; *n* = 12), after 120 (proliferation) or 144 h (IFN-γ) of stimulation with gp43 (1 μg/mL) or CFA (2 or 5 μg/mL), or left untreated (Medium). Results expressed as means with SEM of δ cpm, and capped lines indicate statistically significant differences with respective *p* values * *p* < 0.05, ** *p* < 0.01 or *** *p* < 0.001; black dots above the bars indicate differences in comparison with Medium: • *p* < 0.05 or •• *p* < 0.01.

Previously, gp43 was reported to induce in vitro antigen-specific anergy of T-cell response on PBMC cultures from PCM patients [11]. The CFA, on the other hand, has not yet been reported on human PBMC proliferation assays. Hence, we tested the ability of antigen-presenting cells in PBMCs to stimulate the proliferation of *P. brasiliensis*-specific T-lymphocytes, without the additional presence of DCs loaded with antigens. We investigated the effects of CFA at two different doses (2 and 5 μg/mL) compared with gp43 at 1 μg/mL (determined in a previous study [35]) on cells from controls, and active and treated PCM groups (Figure 4a). PBMCs from the active PCM and control groups did not proliferate with gp43, but showed higher cpm values with CFA at 5 μg/mL compared with those of unstimulated cells (Medium; *p* < 0.01 for both AP and CO). Proliferation on PBMC cultures from all groups was also positively tested with high cpm to PHA (Figure S3a). Regardless, PBMCs from the treated group showed higher proliferation with both antigens (*p* < 0.05 with gp43 and *p* < 0.01 with CFA at 5 μg/mL versus Medium), when compared to that of the CO group (*p* < 0.01 with gp43 and CFA at 2 μg/mL and *p* < 0.001 with CFA at 5 μg/mL), and with gp43 when compared with the active PCM group (*p* < 0.05). We used an ELISA assay to determine the levels of IFN-γ and only found higher levels in PBMCs from the treated patient group, stimulated with CFA at 5 μg/mL, in comparison with Medium or gp43 (Figure 4b). PHA-stimulated PBMCs from PCM patients secreted high levels of IFN-γ, similarly to those from CO (Figure S3b). Supernatants were also assayed for IL-10, but detectable levels were only shown under PHA stimulus (Figure S3c).

### 3.5. P. brasiliensis Antigens-Stimulated MoDCs from PCM Patients Can Induce Strong Proliferative Response

**Figure 5:**
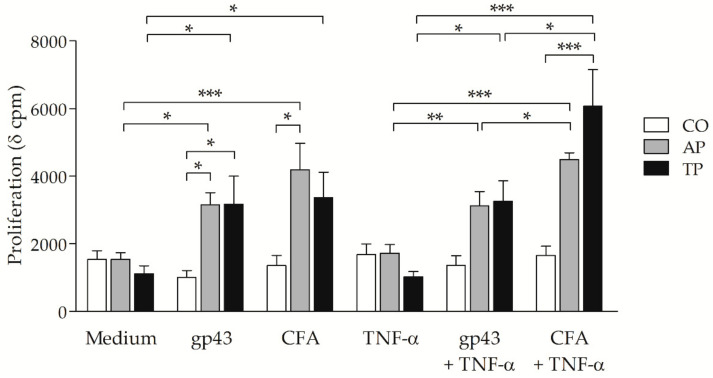
Influence of gp43- and CFA-stimulated moDCs on the proliferative response of autologous lymphocytes: proliferation levels measured by [^3^H]-thymidine uptake on cocultures of autologous lymphocytes and moDCs from non-PCM control subjects (CO: white bars; *n* = 15), and patients with active PCM (AP: grey bars; *n* = 17) or treated PCM (TP: black bars; *n* = 22) previously stimulated with gp43 or CFA, with or without TNF-α, or left untreated (Medium), after 120 h. Results expressed as means with SEM of δ cpm, and capped lines indicate statistically significant differences with respective *p* values: * *p* < 0.05, ** *p* < 0.01, or *** *p* < 0.001.

To assess whether moDCs from patients with PCM could induce antigen-specific lymphoproliferation, we cocultured previously gp43- and CFA-stimulated moDCs, with or without TNF-α, and autologous lymphocytes. Firstly, to rule out any unspecific proliferation, we irradiated all moDC cultures with a ^60^Co source that inactivated any other cell type. We then thawed autologous lymphocytes and adjusted cells into 1:5 proportion (moDCs:lymphocytes) to detect the resulting proliferation after 5 days (Figure 5). The 1:10 and 1:20 ratios were also evaluated, but 1:5 showed higher proliferation levels [36]. Only basal levels of cpm were detected on cultures with irradiated moDCs or lymphocytes on medium, and significantly higher levels of cpm were verified on PHA-cultured lymphocytes, indicating there was no unspecific proliferation and confirming a high viability of thawed cells, respectively (Figure S4).

With or without TNF-α, gp43- and CFA-stimulated moDCs induced higher proliferative response on cells from active and treated PCM groups in comparison to antigen-free moDCs (Medium or TNF-α). Gp43-stimulated moDCs from these groups also led to higher lymphoproliferation when compared with in the CO group. In addition, CFA + TNF-α-stimulated moDCs induced even higher proliferation than that by gp43 + TNF-α on both PCM groups (*p* < 0.05 on both). Importantly, cells from the CO group did not proliferate to *P. brasiliensis* antigens presented by moDCs as observed on PCM groups, confirming specific responses to gp43 and CFA.

### 3.6. CFA-Stimulated MoDCs from PCM Patients Up-Regulated IFN-γ and TNF-α Secretion by Lymphocytes

**Figure 6:**
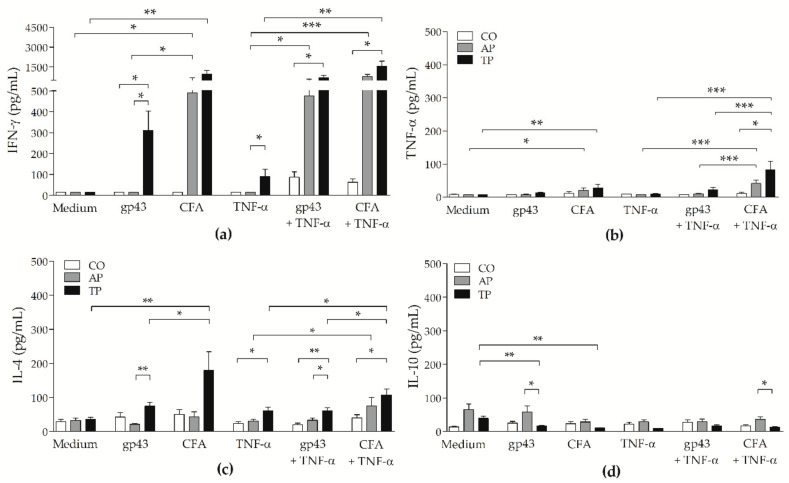
Influence of gp43- and CFA-stimulated moDCs on secretion of cytokines in cocultures with autologous lymphocytes. ELISA-assayed levels of (**a**) IFN-γ, (**b**) TNF-α, (**c**) IL-4 and (**d**) IL-10 (pg/mL) were measured on cocultures of autologous lymphocytes and moDCs of non-PCM control subjects (CO: white bars; *n* = 15), and patients with active PCM (AP: grey bars; *n* = 17) or treated PCM (TP: black bars; *n* = 22) previously stimulated with gp43 or CFA, with or without TNF-α, or left untreated (Medium), after 144 h. Results expressed as means with SEM of levels, and capped lines indicate statistically significant differences with respective *p* values: * *p* < 0.05, ** *p* < 0.01, or *** *p* < 0.001.

In addition to the proliferative response, we investigated the levels of IFN-γ, TNF-α, IL-4 and IL-10 in supernatants from moDCs + lymphocytes cocultures by ELISA assay. In the cultures from control, active and treated PCM groups, only basal levels of these cytokines were detected in cultures of irradiated moDCs or lymphocytes in medium, and significantly higher levels of IFN-γ, TNF-α, and IL-10 were verified in PHA-cultured lymphocytes compared with in lymphocytes in medium (Figure S5). Increased levels of IL-4 on moDCs + Medium from all groups were likely a result of recombinant IL-4 added for the differentiation of monocytes into DCs (Figure S5c). Significantly upregulated levels of IFN-γ and TNF-α were detected on cocultured lymphocytes and CFA- and CFA + TNF-α-stimulated moDCs from the active and treated PCM groups in contrast to antigen-free moDCs (Medium or TNF-α; Figure 6a,b, respectively). However, this effect was more prominent on treated PCM. Gp43 + TNF-α-stimulated moDCs induced the secretion of IFN-γ on PCM patients’ cells, but had little effect on TNF-α levels. CFA-stimulated moDCs from the treated patients led to higher levels of IL-4 compared with those of unstimulated cells or with gp43-stimulated moDCs (Figure 6c). On the active PCM group, there were higher levels of IL-10 on cocultures of gp43- and CFA + TNF-α-stimulated moDCs and lymphocytes in comparison with those of treated patient cells. Conversely, gp43- and CFA-stimulated moDCs from the treated PCM patients, without TNF-α, inhibited IL-10 secretion (Figure 6d).

## 4. Discussion

Great advances have been achieved in the knowledge of host–parasite interactions in PCM, but several mechanisms involving the fate of infection and its progression to disease in its different clinical manifestations in humans are not known [37,38]. Additionally, studies sought new ways to modulate the cellular immune response to confer greater resistance to the host in various mycoses [39,40]. As the most effective APCs, DCs could be used to modulate immune responses of lymphocytes against a specific antigenic fraction. In this context, our study investigated monocyte-derived DCs from patients with PCM and their role in adaptive immune response.

Considering that the antigen presentation and the induction of a T-cell response by DCs depend on the maturation of these cells, represented by MHC-II and costimulatory molecules expression, we observed high expression of HLA-DR and CD86 on moDCs from patients with PCM, especially with TNF-α. These results showed that moDCs from patients with active or treated PCM can be differentiated in vitro, and express surface molecules similarly or higher than that on cells from non-PCM control subjects (CO). Percentages of CD80^+^ cells were higher in moDCs from patients with active PCM (AP), but in general, frequencies of this molecule were low in all groups, with gp43 significantly decreasing the expression of this molecule in moDCs from patients with treated PCM (TP). Corroborating our findings, the stimulation of gp43 was associated with the low expression of MHC-II, CD86, and CD80 on DCs from *P. brasiliensis*-infected mice, suggesting a similar inhibitory effect of this antigen on human moDCs [17,18]. We did not find differences in the frequencies or MFI of DC-SIGN on moDCs from patients with PCM; nevertheless, the previously reported recognition of the polysaccharide portion of extracellular vesicles from *P. brasiliensis* by DC-SIGN encourages future studies with different antigens [41]. These vesicles constitute an unconventional transport of molecules through the fungal cell wall, but binding with DC-SIGN could have a role in facilitating phagocytosis by DCs.

In addition, our results showed that moDCs from treated patients release considerably higher doses of IL-12p40 than cells from patients with active PCM or from the CO group do, mainly when stimulated with TNF-α. DCs are a major source of IL-12, which is considered crucial in both human and experimental PCM, leading to a protective Th1 immune response [9,42,43]. Although the bioactive form is IL-12p70, the p40 subunit of IL-12 was associated with resistance to PCM [44], and found in great amounts during the late periods of *P. brasiliensis* infection [45]. We cannot ruled out the possibility of having measured both IL-12 and IL-23, which share the p40 subunit. IL-23 is also secreted by APCs and its production is mainly activated by the binding of β-glucans on *P. brasiliensis* cell wall to dectin-1, a pattern recognition receptor expressed by DCs and other cell types [46]. This cytokine promotes the maturation of Th17 cells expressing IL-17, and Th22 cells expressing IL-22, resulting in chronic tissue inflammation. Because the great majority of patients in this study had the chronic multifocal form of PCM (86.8%), the presence of both IL-12 and IL-23 is expected, given that these cytokines lead to a mixed immune response of Th1, Th17 and Th22, observed in this form of the disease [10]. Furthermore, our findings demonstrated that moDCs do not lose the potential of inducing an IL-12p40-associated immune response even during active PCM, with similar levels of this cytokine to those of the control group.

Although IL-1β induces increased secretion of IL-12 by DCs in vitro [47], in our study, this cytokine was detected at low levels in moDCs from all groups. However, considering the essential role of IL-1β on PCM and the recent findings on the nucleotide-binding oligomerization domain(NOD)-like receptor P3 (NLRP3) inflammasome on DCs [24], further investigation is required. Likewise, the levels of IL-10 that we observed were low and apparently downregulated by TNF-α. Although chemokine CCL18 is induced by IL-10 on DCs [48], there were no differences in the levels of the latter that could be associated with substantial amounts of the former on active PCM patient cells in comparison with those of treated ones.

Remarkably, our study uncovered that moDCs from the active PCM group secreted larger amounts of CCL18 than those from treated patients in all evaluated conditions, and it may be a beneficial function of moDCs during active disease, as the main role of this chemokine is to attract naïve cells. This chemokine could also be induced through Pattern Recognition Receptors signaling, considering that CFA stimulated a high production of CCL18 by moDCs from non-PCM subjects. CFA is an antigen compound of various molecules, such as the 75 kDa glycoprotein, which is immunogenic and contains a homologous amino acid sequence (87%) to other fungi such as *Aspergillus fumigatus* and *Coccidioides immitis* [49]. CCL18 is constitutively expressed by DCs at high levels, with preferential attraction of naïve T cells, B cells, and immature moDCs, reported mainly in autoimmune disorders and other chronic inflammatory [50,51] and infectious [48,52,53,54,55] diseases. A protective role of CCL18 was shown in experimental malaria [52], and was associated with the maintenance of chronic inflammation in the lungs and liver, during tuberculosis and hepatitis C, respectively [53,54]. Contact with *Candida albicans* downregulated CCL18 on DCs and in the coculture of epithelial cells, moDCs and *Aspergillus fumigatus* germ tubes, the expression of this chemokine gene had a 2.377-fold change [48,55]. On the other hand, CCL18 was also reported as a major inducer of pulmonary cystic fibrosis and emphysema [56,57]. Considering that 75.5% of patients with PCM in this study had *P. brasiliensis*-related lesions in their lungs, our data suggest that this chemokine may be associated with the respiratory abnormalities frequently observed in these patients.

Recently, Amorim et al. investigated the role of NLRP3 in human PCM and found that hypoxic cells from patients, which are characterized by the intracellular expression of hypoxia-inducible transcription factor (HIF)-1α, had higher activation of this inflammasome than the control subjects did [37]. Typically, inflamed tissue has low levels of oxygen, triggering the HIF-1α that up-regulates the *IL12B* gene to produce more IL-12p40, and inducing Th2 cytokines profile in DCs, which, in turn, favors the secretion of CCL18 [58,59]. Therefore, during active pulmonary chronic PCM, moDCs could have a high expression of HIF-1α and NLRP3, with consequential up-regulation of IL-1β that favors fibrosis and could induce the Th22 response, with IL-21 and IL-23. In parallel, an increase in IL-18 may occur, which leads to either a Th1 response with IL-12 or Th17 response with IL-23, also associated with pulmonary fibrosis [56]. In addition, large amounts of CCL18 induce the production of collagen by pulmonary fibroblasts [50], accompanied by high levels of profibrotic growth factors, transforming growth factor beta(TGF)-β and basic fibroblast growth factor (bFGF) [27]. With treatment, lesions ameliorate, but this does not mean full recovery of respiratory functions. The resulting sequelae, such as emphysema and fibrosis, can result in chronic hypoxia that maintains the expression of HIF-1α, decreasing CCL18 [59] and up-regulating IL-12p40.

Our results on PBMCs showed T-cell anergy from active PCM patients, with low IFN-γ, but undetectable IL-10 in all groups. Previous studies also reported the gp43-induced antigen-specific anergy of T-cell response in PBMC cultures, with low levels of IFN-γ but high IL-10. After treatment, PBMCs proliferate with gp43, with high levels of both cytokines [11,60]. The increased levels of IL-10 on those previous reports may be the result of the concentration of gp43, which was substantially higher than the 1 μg/mL used in the present study. Moreover, in PBMC cultures from resistant mice, CFA from the *P. brasiliensis* 18 strain induced high lymphoproliferation, with high IFN-γ levels in the early phase of infection [7]. Similarly, PBMCs from patients with the active disease had increased proliferation with CFA; however, we prepared this antigen with the *P. brasiliensis* B-339 strain that has higher gp43 content and other immunogenic glycoproteins when compared with the 18 strain [61], which resulted in higher levels of IFN-γ only in cells from the treated group. We confirmed gp43 in the CFA from the *P. brasiliensis* B-339 strain by electrophoresis [36], similar to a previous report [30]. The presence of this glycoprotein in a more complex antigenic compound may be crucial to effects observed in our study, as the immunization with a gp43-free CFA from the *P. brasiliensis* 18 strain, followed by infection with *P. brasiliensis* yeast induced great severity in PCM [62].

Standing as novel findings, moDCs were able to revert the in vitro antigen-specific anergy of T cells during the active disease, and gp43 presentation induced higher proliferation of autologous lymphocytes in comparison with control subject cells, but only up-regulated IFN-γ with the additional stimulus of TNF-α. CFA-stimulated moDCs had more prominent effects. They induced higher proliferation of lymphocytes from both active and treated groups in comparison with the controls, and with gp43-stimulated moDCs, also upregulating IFN-γ and TNF-α, corroborating results obtained in coccidioidomycosis [34]. In addition, IL-4 was higher in the treated PCM group, but with simultaneously higher levels of TNF-α and IFN-γ. This concomitant production of IL-4 and IFN-γ reported in untreated patients with the chronic multifocal form of PCM [10,63]. Hence, the present finding may reflect the tendency of treated PCM towards the Th1 response, but with IL-4 secretion, possibly explaining the long-term treatment with frequent relapses of patients with this form of the disease. It may also represent traces of untreated Th0 clones, secreting both cytokines, as described in untreated tuberculosis in comparison with Th1 clones in treated patients [64]. In spite of increased IL-4, the levels of IL-10 were downregulated by gp43 and CFA in the cocultures from the treated patients group, which was beneficial in the murine model of PCM. The absence or inactivation of IL-4 did not protect susceptible mice in *P. brasiliensis* infection, whereas the absence of IL-10 was associated with a favorable immune response and pathogenicity regression [65,66].

The different effects of antigen presentation by moDCs became even more evident when comparing the proliferative responses to gp43 and CFA in PBMC assays and in cocultures with autologous lymphocytes. Gp43 alone induced high proliferation only in PBMCs from the treated PCM group, whereas, with moDCs, there was higher proliferation in both groups of patients compared with that in the control group. CFA, on the other hand, induced lymphoproliferation in assays with PBMCs from all groups, but only cells from the treated patients significantly proliferated more than those from the control subjects. Importantly, in the cocultures, CFA-stimulated moDCs induced significant proliferation of cells from both groups of patients, but not of the non-PCM control group.

In our study, patients with PCM were evaluated and divided into two groups of active and cured disease, as described in Section 2. In analysis according to acute and chronic (uni- and multifocal) forms, no statistically significant differences were observed between groups, and the analysis with male patients only showed similar results to those from the analysis with both sexes [36]. Future studies with larger numbers of patients, mainly with acute and chronic unifocal forms, and patients with PCM under immunosuppression could enable further discussion on the role of DCs in severe and milder forms. In addition, a follow-up study of patients with PCM before, during, and after treatment could complement current knowledge on the variable immune responses along the evolution of this mycosis. Possible limitations in our study are the lack of evaluation of antigen effects on moDC innate mechanisms, on the modulation of other T-helper responses such as Th17 and Th22 by moDCs, and on the lymphocyte expression of costimulatory molecules or cytokine receptors.

In addition to conventional treatment with antifungal drugs, interest in alternative therapies has greatly increased in recent years, seeking the reduction of drug toxicity and improving survival in severe and fatal cases of this mycosis. In this context, significant advances were made with the use of DCs as prophylactic and therapeutic vaccines in experimental PCM [67,68].

Many questions still need answers to enable the development of more efficient and protective immune responses, such as the use of DCs in immunotherapy on human PCM. As demonstrated in our study, different antigenic fractions of *P. brasiliensis* can modulate DCs and induce various immune responses in cells from patients. As an additional new finding, we showed DCs’ capacity to revert the characteristic in vitro immunosuppression of this disease. Hence, our study raises new prospects for the use of DCs as tools for choosing suitable fungal antigens, with subsequent analyses of immune responses and their use in immunotherapy in human PCM.

## Data Availability

The data presented in this study are available on request from the corresponding author. The data are not publicly available due ethical restrictions.

## Supplementary Materials

The following are available online at https://www.mdpi.com/2309-608X/7/3/201/s1, Table S1: Characteristics of Active and Treated PCM Patients and Non-PCM Control Subjects, Table S2a–d: Statistical Summary, Figure S1: Gating strategy for Flow Cytometric Analyses, Figure S2: Influence of gp43 and CFA of *P. brasiliensis* on the Expression of Surface Molecules by MoDCs, Figure S3: Induction of Lymphoproliferation and Secretion of IFN-γ and IL-10 by PHA on PBMC Cultures, Figure S4: Control Cultures for the Analysis of the Proliferative Response of Autologous Lymphocytes, Figure S5: Cytokines on Control Cultures of MoDCs or Autologous Lymphocytes.
