# Supplementary material for: Monocyte-Derived Dendritic Cells Can Revert In Vitro Antigen-Specific Cellular Anergy in Active Human Paracoccidioidomycosis
Source: J Fungi (Basel). 2021 Mar 10;7(3):201. doi: 10.3390/jof7030201 (PMC8000053; doi:10.3390/jof7030201)
Supplement: Supplementary file 1 [file jof-07-00201-s001.pdf]

Journal of *Fungi*

MDPI

Article – Supplementary Material

Monocyte-Derived Dendritic Cells Can Revert In Vitro Antigen-Specific Cellular Anergy In Active Paracoccidioidomycosis

Table S1 – Characteristics of Active and Treated PCM Patients and Non-PCM Control Subjects. Demographic, clinical and laboratorial aspects.

Characteristics		Groups						
		Control	Active PCM			Treated PCM		
			Acute	Multifocal Chronic	Unifocal Chronic	Acute	Multifocal Chronic	Unifocal Chronic
**Sex** — n (%)	Male	21 (70.0)	2 (100.0)	20 (95.2)	1 (100.0)	3 (100.0)	21 (84.0)	1 (100.0)
	Female	9 (30.0)	0 (0.0)	1 (4.8)	0 (0.0)	0 (0.0)	4 (16.0)	0 (0.0)
			24 (45.3)			29 (54.7)		
	Total — n	30	53					
**Age** — Mean (± SD)[a]		40 (± 18.4)	51 (± 13.1)			52 (± 12.6)		
**CIE (1:)** — Median (Interquartile Range)[b]			32 (16 – 64)			2 (NR – 2)		
**Affected sites** — n (%)	Lungs		1 (50.0)	17 (70.8)[c]	0	0	22 (75.9)[c]	0
	Oral mucosa, pharynx or larynx		1 (50.0)	14 (56.0)	1 (100.0)	0	11 (37.9)	0
	Lymphadenomegaly		1 (50.0)	10 (40.0)	0	3 (100.0)	9 (31.0)	0
	Bronchus or Trachea		0	2 (8.0)	0	0	1 (3.5)	0
	Central Nervous System		0	2 (8.0)	0	0	2 (6.9)	0
	Adrenals		0	1 (4.0)	0	0	0	1 (100.0)
	Skin		1 (50.0)	5 (20.0)	0	0	3 (10.3)	0

a. Mean values of age in years ± Standard Deviation; b. CIE = Counterimmunoelectrophoresis titers; c. Patients with affected lungs (Active + Treated PCM): 75.5% (40 of 53).

Table S2a – Statistical Summary. Statistically significant differences in the analyses of variables (percentage and Mean Fluorescence Intensity of positively stained cells, proliferation of autologous lymphocytes and levels of cytokines) on moDCs from patients with PCM and control subjects; comparisons between groups in each culture by ANOVA and post-test of Bonferroni, with respective *p* values.

Cultures	Variable	Compared Groups		ANOVA *p* values	Post-test *p* values
Medium	% HLA-DR$^+$	Active PCM	Treated PCM	0.0266	< 0.05
	% CD86$^+$	Control	Active PCM	0.0103	< 0.05
		Control	Treated PCM		< 0.05
	IL-12p40	Active PCM	Treated PCM	0.0206	< 0.05
	CCL18	Control	Active PCM	0.0080	< 0.05
		Active PCM	Treated PCM		< 0.05
gp43	% HLA-DR$^+$	Active PCM	Treated PCM	0.0393	< 0.05
	HLA-DR MFI[a]	Active PCM	Treated PCM	0.0189	< 0.05
	% CD86$^+$	Control	Treated PCM	0.0046	< 0.01
	% CD80$^+$	Active PCM	Treated PCM	0.0222	< 0.05
	IL-12p40	Active PCM	Treated PCM	0.0234	< 0.05
	CCL18	Control	Active PCM	0.0053	< 0.05
		Active PCM	Treated PCM		< 0.01
	Lymphoproliferation	Control	Active PCM	0.0151	< 0.05
		Control	Treated PCM		< 0.05
	IFN-γ	Control	Treated PCM	0.0089	< 0.05
		Active PCM	Treated PCM		< 0.05
	IL-4	Active PCM	Treated PCM	0.0058	< 0.01
	IL-10	Active PCM	Treated PCM	0.0334	< 0.05
CFA	% CD86$^+$	Control	Treated PCM	0.0338	< 0.05
	IL-12p40	Control	Treated PCM	0.0129	< 0.05
		Active PCM	Treated PCM		< 0.05
	CCL18	Control	Treated PCM	0.0014	< 0.05
		Active PCM	Treated PCM		< 0.01
	Lymphoproliferation	Control	Active PCM	0.0409	< 0.05
TNF-α	HLA-DR MFI[a]	Active PCM	Treated PCM	0.0228	< 0.05
	CD86 MFI[a]	Active PCM	Treated PCM	0.0327	< 0.05
	% CD80$^+$	Control	Active PCM	0.0007	< 0.01
		Active PCM	Treated PCM		< 0.001
	IL-12p40	Control	Treated PCM	< 0.0001	< 0.001
		Active PCM	Treated PCM		< 0.001
	CCL18	Active PCM	Treated PCM	0.0239	< 0.05
	IFN-γ	Active PCM	Treated PCM	0.0144	< 0.05
	IL-4	Control	Treated PCM	0.0157	< 0.05
gp43 + TNF-α	HLA-DR MFI[a]	Active PCM	Treated PCM	0.0336	< 0.05
	% CD80$^+$	Control	Active PCM	0.0001	< 0.01
		Active PCM	Treated PCM		< 0.001
	IL-12p40	Control	Treated PCM	< 0.0001	< 0.01
		Active PCM	Treated PCM		< 0.001
	CCL18	Active PCM	Treated PCM	0.0123	< 0.05
	IFN-γ	Control	Treated PCM	0.0138	< 0.05
	IL-4	Control	Treated PCM	0.0035	< 0.01
		Active PCM	Treated PCM		< 0.05
CFA + TNF-α	% CD80$^+$	Control	Treated PCM	0.0376	< 0.05
	IL-12p40	Control	Treated PCM	0.0007	< 0.01
		Active PCM	Treated PCM		< 0.01
	CCL18	Control	Treated PCM	0.0025	< 0.05
		Active PCM	Treated PCM		< 0.01
	Lymphoproliferation	Control	Treated PCM	0.0013	< 0.001
	IFN-γ	Control	Treated PCM	0.0282	< 0.05
	TNF-α	Control	Treated PCM	0.0154	< 0.05
	IL-4	Control	Treated PCM	0.0451	< 0.05
	IL-10	Active PCM	Treated PCM	0.0146	< 0.05

a. MFI: Mean Fluorescence Intensity.

Table S2b – Statistical Summary. Statistically significant differences in the analyses of variables (percentage and Mean Fluorescence Intensity of positively stained cells, proliferation of autologous lymphocytes and levels of cytokines) on moDCs from patients with PCM and control subjects; comparisons between cultures within each group by ANOVA and post-test of Bonferroni, with respective *p* values.

Group	Variable	Compared Cultures		ANOVA *p* values	Post-test *p* values
**Control**	IL-12p40	Medium	gp43	0.0274	< 0.05
	CCL18	Medium	CFA	0.0140	< 0.01
		gp43	CFA		< 0.05
**Active PCM**	IL-12p40	TNF-α	CFA + TNF-α	0.0342	< 0.05
	Lymphoproliferation	Medium	gp43	0.0007	< 0.05
		Medium	CFA		< 0.001
		TNF-α	gp43 + TNF-α	< 0.0001	< 0.01
		TNF-α	CFA + TNF-α		< 0.001
		gp43 + TNF-α	CFA + TNF-α		< 0.05
	IFN-γ	Medium	CFA	0.0069	< 0.05
		gp43	CFA		< 0.05
		TNF-α	gp43 + TNF-α	0.0004	< 0.05
		TNF-α	CFA + TNF-α		< 0.001
	TNF-α	Medium	CFA	0.0258	< 0.05
		TNF-α	CFA + TNF-α	<0.0001	< 0.001
		gp43 + TNF-α	CFA + TNF-α		< 0.001
	IL-4	TNF-α	CFA + TNF-α	0.0313	< 0.05
**Treated PCM**	% CD80+	gp43 + TNF-α	CFA + TNF-α	0.0218	< 0.05
		Medium	gp43	0.0074	< 0.01
	Lymphoproliferation	Medium	gp43	0.0123	< 0.05
		Medium	CFA		< 0.05
		TNF-α	gp43 + TNF-α	<0.0001	< 0.05
		TNF-α	CFA + TNF-α		< 0.001
		gp43 + TNF-α	CFA + TNF-α		< 0.05
	IFN-γ	Medium	CFA	0.0063	< 0.01
		TNF-α	CFA + TNF-α	0.0035	< 0.01
	TNF-α	Medium	CFA	0.0094	< 0.01
		TNF-α	CFA + TNF-α	< 0.0001	< 0.001
		gp43 + TNF-α	CFA + TNF-α		< 0.001
	IL-4	Medium	CFA	< 0.0001	< 0.01
		gp43	CFA		< 0.05
		TNF-α	CFA + TNF-α	0.0223	< 0.05
		gp43 + TNF-α	CFA + TNF-α		< 0.05
	IL-10	Medium	gp43	0.0003	< 0.01
		Medium	CFA		< 0.01

Table S2c – Statistical Summary. Statistically significant differences in the analyses of variables (lymphoproliferation and levels of IFN-γ) on PBMCs from patients with PCM and control subjects; comparisons between groups in each culture by ANOVA and post-test of Bonferroni, with respective *p* values.

Cultures	Variable	Compared Groups		ANOVA *p* values	Post-test *p* values
gp43	Lymphoproliferation	Control	Treated PCM	0.0041	< 0.01
		Active PCM	Treated PCM		< 0.05
CFA 2 μg/mL	Lymphoproliferation	Control	Treated PCM	0.0048	< 0.01
CFA 5 μg/mL	Lymphoproliferation	Control	Treated PCM	0.0011	< 0.001
	IFN-γ	Active PCM	Treated PCM	0.0207	< 0.05

Table S2d – Statistical Summary. Statistically significant differences in the analyses of variables (lymphoproliferation and levels of IFN-γ) on PBMCs from patients with PCM and control subjects; comparisons between cultures within each group by ANOVA and post-test of Bonferroni, with respective *p* values.

Group	Variable	Compared Cultures		ANOVA *p* values	Post-test *p* values
Control	Lymphoproliferation	Medium	CFA 2 μg/mL	< 0.0001	< 0.05
		Medium	CFA 5 μg/mL		< 0.001
Active PCM	Lymphoproliferation	Medium	CFA 5 μg/mL	0.0011	< 0.01
		gp43	CFA 5 μg/mL		< 0.01
Treated PCM	Lymphoproliferation	Medium	gp43	0.0096	< 0.05
		Medium	CFA 5 μg/mL		< 0.01
	IFN-γ	Medium	CFA 5 μg/mL	0.0038	< 0.01
		gp43	CFA 5 μg/mL		< 0.05

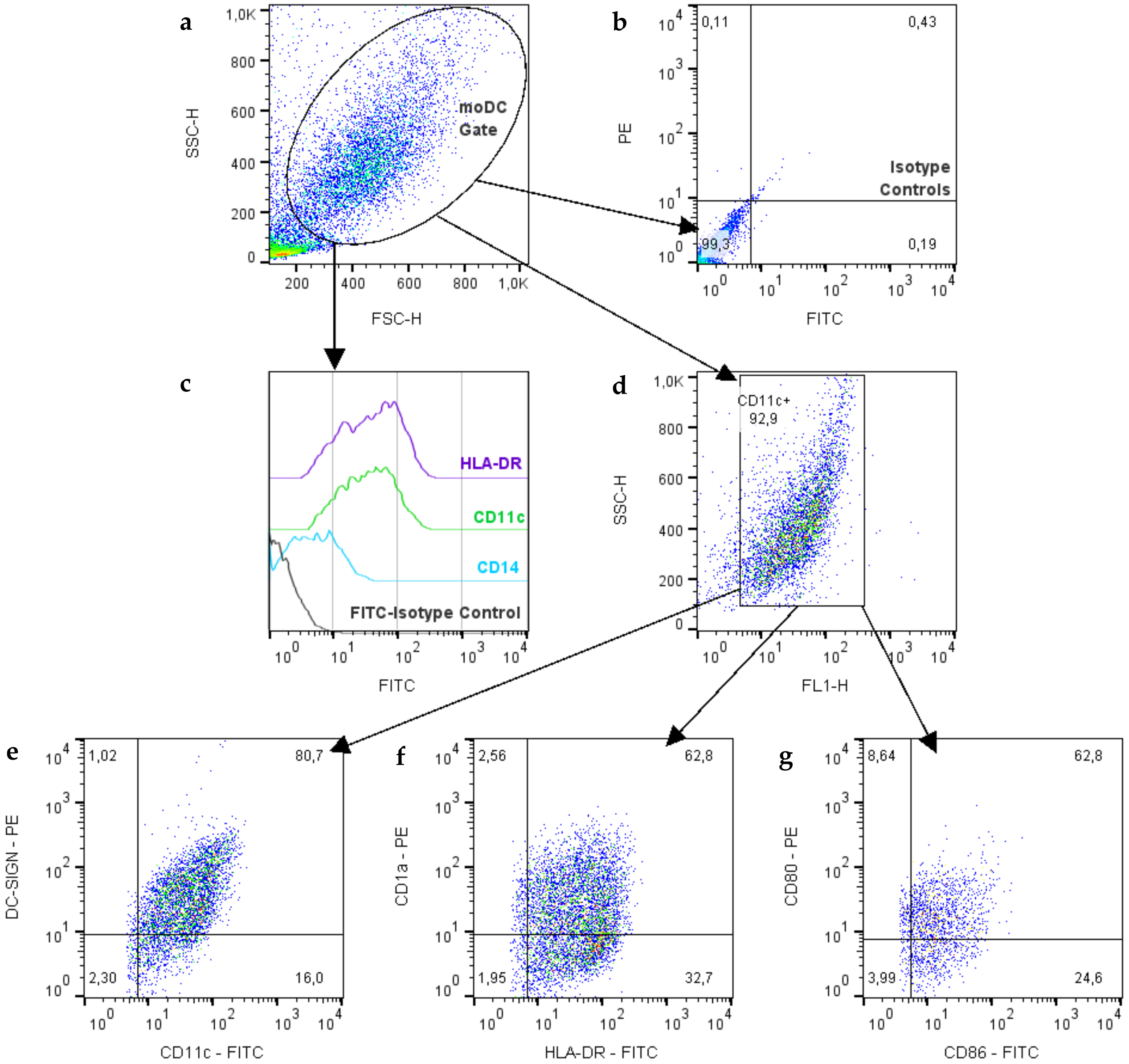

Figure S1. Gating strategy for Flow Cytometric Analyses. Monocyte-derived DCs were generated *in vitro* and stimulated for 48 h with *Paracoccidioides brasiliensis* antigens, gp43 or CFA, with or without TNF-α activation, or left untreated. Cells were stained with specific mAbs, and then acquired and analyzed on a FACSCalibur flow cytometer and CellQuest software (BD Biosciences, San Jose, CA, USA) for (**a**) size (SSC) and granularity (FSC), and a gate of moDCs was created accordingly. Gated moDCs were stained with (**b**) specific mouse isotype controls for FITC and PE, and (**c**) moDCs were determined as negatively stained CD14-, and positively stained CD11c- (or CD1a; data not shown) and HLA-DR-cells; (**d**) a second gate of CD11c+ moDCs was determined and used to analyze the expression of (**e**) CD11c and DC-SIGN, (**f**) HLA-DR and CD1a, and (**g**) CD86 and CD80. Plots are representative results of TNF-α-activated moDCs from one patient with active PCM.

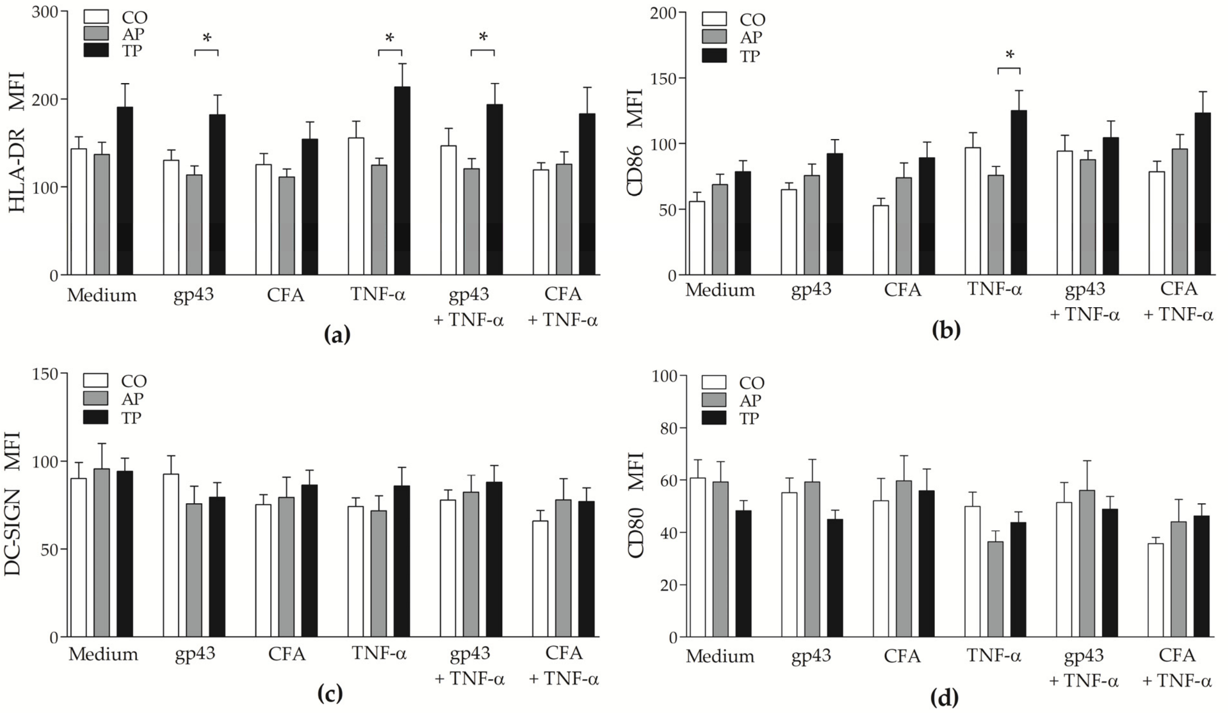

Figure S2. Influence of gp43 and CFA of *P. brasiliensis* on the Expression of Surface Molecules by MoDCs. Medium Fluorescence Intensity (MFI) of **(a)** HLA-DR, **(b)** CD86, **(c)** DC-SIGN, and **(d)** CD80 molecules were analyzed by flow cytometry on gated MoDCs from non-PCM control subjects (CO: white bars; n = 15), and patients with active PCM (AP: grey bars; n = 17) or with treated PCM (TP: black bars; n = 22), after 48 h of incubation without stimulus (Medium) or with gp43 or CFA, with or without TNF-α. Results are expressed as mean with SEM of MFI: *$p < 0.05$ between groups.

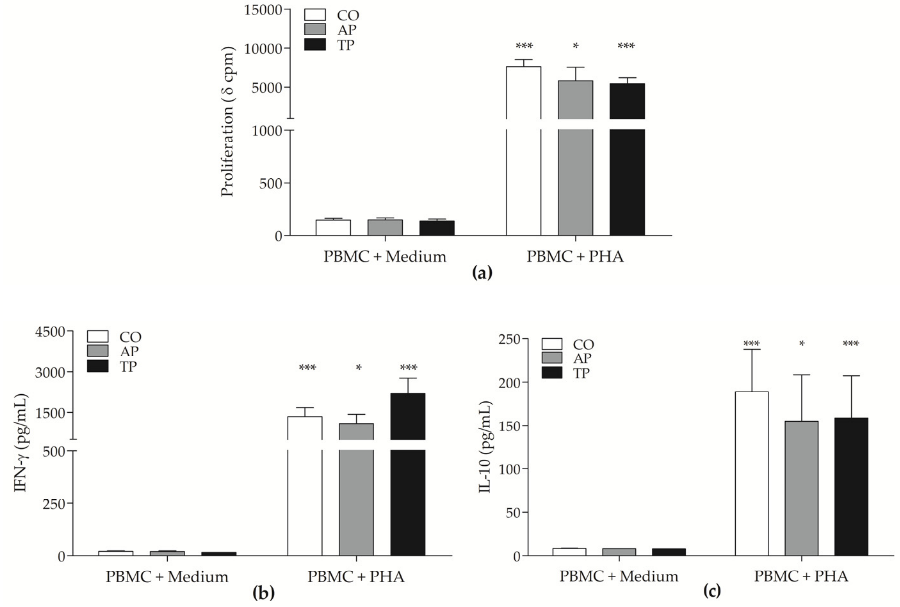

Figure S3. Induction of Lymphoproliferation and Secretion of IFN-γ and IL-10 by PHA on PBMC Cultures. (a) Proliferation of PBMCs was measured by [$_3$H]-thymidine uptake on cells, **(b)** levels of IFN-γ (pg/mL) and **(c)** IL-10 (pg/mL) were determined on culture supernatants from non-PCM control subjects (CO: white bars; n = 17), and patients with active PCM (AP: grey bars; n = 8) or with treated PCM (TP: black bars; n = 12), after 120 (proliferation) or 144 h (cytokines) of incubation without stimulus (PBMC+Medium) or with PHA (PBMC+PHA). Results are expressed as mean with SEM of δ cpm (a) and levels (b,c): *$p < 0.05$ or ***$p < 0.001$ versus PBMC+Medium.

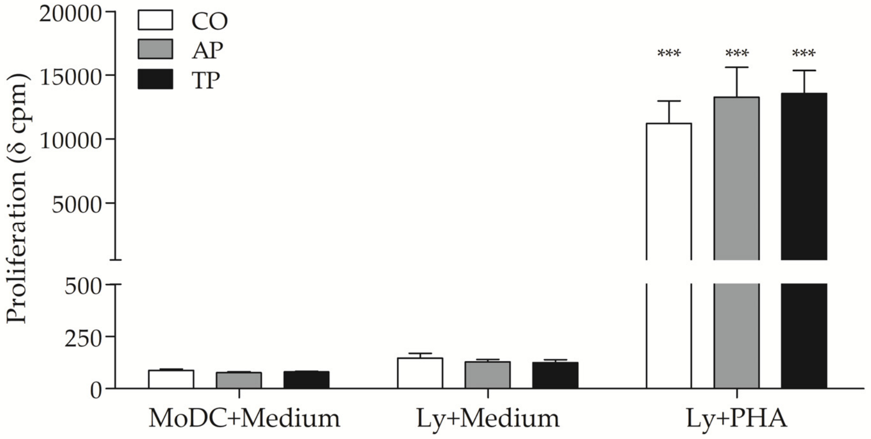

Figure S4. Control Cultures for the Analysis of the Proliferative Response of Autologous Lymphocytes. The proliferation levels were measured by [3H]-thymidine uptake on cultures of unstimulated moDCs (MoDC+Medium), autologous lymphocytes (Ly) without stimulus (Ly+Medium) or with PHA (Ly+PHA) from non-PCM control subjects (CO: white bars; n = 15), and patients with active PCM (AP: grey bars; n = 17) or with treated PCM (TP: black bars; n = 22), after 120 h. Results are expressed as mean with SEM of δ cpm: ***$p < 0.001$ Ly+PHA versus Ly+Medium.

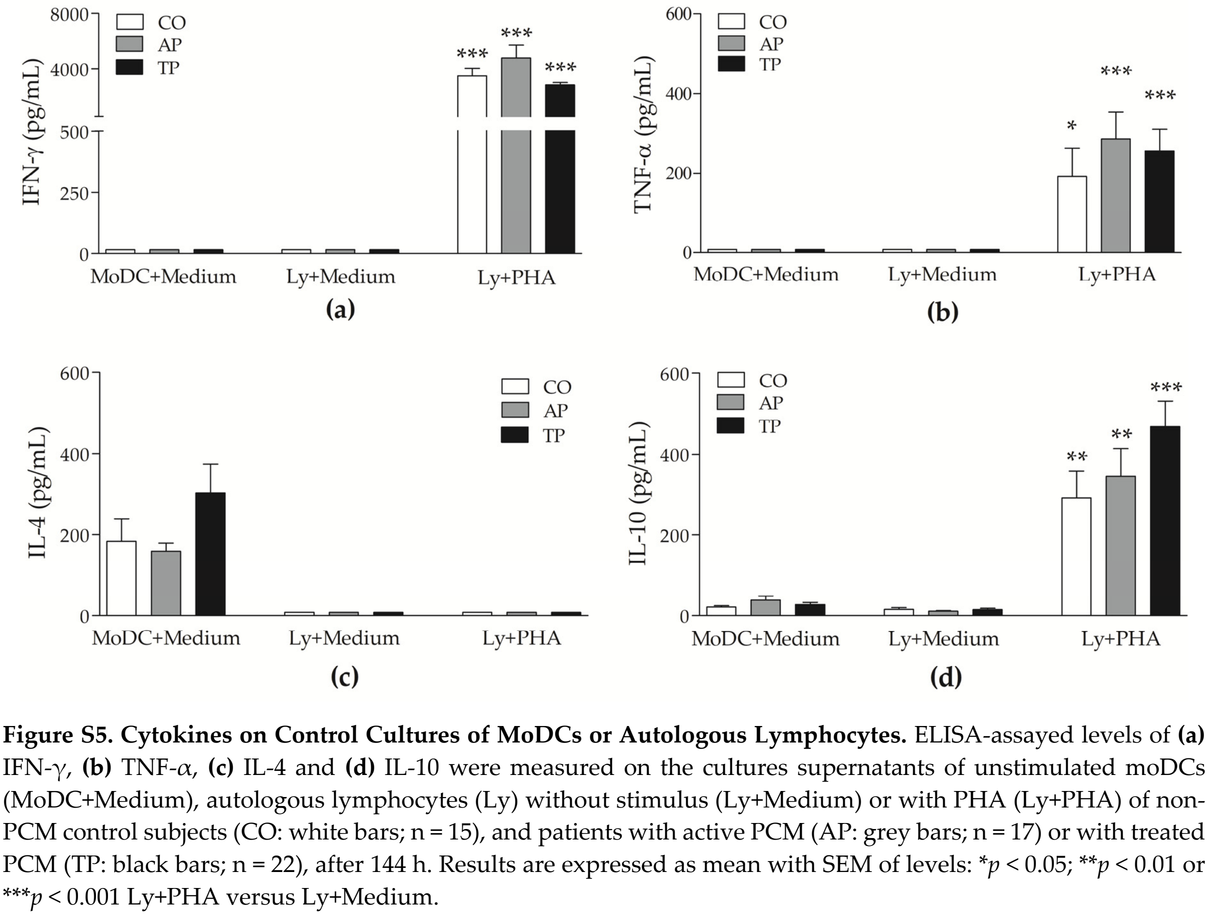

Figure S5. Cytokines on Control Cultures of MoDCs or Autologous Lymphocytes. ELISA-assayed levels of **(a)** IFN-γ, **(b)** TNF-α, **(c)** IL-4 and **(d)** IL-10 were measured on the cultures supernatants of unstimulated moDCs (MoDC+Medium), autologous lymphocytes (Ly) without stimulus (Ly+Medium) or with PHA (Ly+PHA) of non-PCM control subjects (CO: white bars; n = 15), and patients with active PCM (AP: grey bars; n = 17) or with treated PCM (TP: black bars; n = 22), after 144 h. Results are expressed as mean with SEM of levels: *$p < 0.05$; **$p < 0.01$ or ***$p < 0.001$ Ly+PHA versus Ly+Medium.
